# Protocol for a randomised controlled trial of risk screening and early intervention comparing child- and family-focused cognitive-behavioural therapy for PTSD in children following accidental injury

**DOI:** 10.1186/1471-244X-10-92

**Published:** 2010-11-16

**Authors:** Justin Kenardy, Vanessa Cobham, Reginald DV Nixon, Brett McDermott, Sonja March

**Affiliations:** 1 Centre of National Research on Disability and Rehabilitation Medicine, School of Medicine, University of Queensland, Herston QLD 4029, Australia; 2 School of Psychology, University of Queensland, St Lucia QLD 4072, Australia; 3 Child and Youth Mental Health Service, Mater Children's Hospital, Annerley Road, South Brisbane QLD 4101, Australia; 4 School of Psychology, Flinders University, Adelaide SA 5001, Australia

## Abstract

**Background:**

Accidental injury represents the most common type of traumatic event to which a child or adolescent may be exposed, with a significant number of these children going on to experience posttraumatic stress disorder (PTSD). However, very little research has examined potential interventions for the treatment of PTSD in these children. The present trial aims to evaluate and compare child- and family-focused versions of a cognitive-behavioural early intervention for PTSD following accidental injury.

**Methods/Design:**

The principal clinical question under investigation is the efficacy of an early, trauma-focused cognitive-behavioural intervention for the treatment of PTSD in children following accidental injury. Specifically, we compare the efficacy of two active treatments (child-focused and family-focused CBT) and a waitlist control (no therapy) to determine which is associated with greater reductions in psychological and health-related outcome measures over time. The primary outcome will be a reduction in trauma symptoms on a diagnostic interview in the active treatments compared to the waitlist control and greater reductions in the family-compared to the child-focused condition. In doing so, this project will also trial a method of stepped screening and assessment to determine those children requiring early intervention for PTSD following accidental injury.

**Discussion:**

The present trial will be one of the first controlled trials to examine a trauma-focused CBT, early intervention for children experiencing PTSD following accidental injury (as opposed to other types of traumatic events) and the first within a stepped care approach. In addition, it will provide the first evidence comparing the efficacy of child and family-focused interventions for this target group. Given the significant number of children and adolescents exposed to accidental injury, the successful implementation of this protocol has considerable implications. If efficacious, this early intervention will assist in reducing symptoms of traumatic stress as well as preventing chronic disorder and disability in children experiencing acute PTSD following accidental injury.

**Trial Registration:**

Controlled-trials.com: ISRCTN79049138

## Background

Posttraumatic Stress Disorder (PTSD) is an anxiety disorder that can develop following exposure to various traumatic events. It consists of three core symptom clusters; re-experiencing of a traumatic event, emotional numbing or avoidance of reminders of that event and physiological hyperarousal. Children and adolescents may experience PTSD following exposure to a wide variety of traumatic events, including sexual or physical abuse, war, natural disasters, accidents and medical-related traumas. The trauma that triggers the development of PTSD may be either of a recurring nature (e.g., ongoing sexual or physical abuse), or it may be a single incident trauma (e.g., exposure to natural disaster, motor vehicle accident (MVA) or injury).

Approximately 25% of children and adolescents report experiencing a significant traumatic event by the age of 16 years [1]. In Australia, accidental injuries (e.g. bicycle accidents, burns, sporting injuries) represent the most common type of traumatic event experienced by youth, with approximately 2,500 per 100,000 (2.5%) children and adolescents experiencing a serious accidental injury necessitating a hospital admission each year [2]. Although the majority of youth demonstrate great resilience or appear to be only briefly affected by such traumatic events, a significant minority of young people will develop PTSD or other psychological difficulties following exposure to a traumatic event or ongoing trauma. A recent meta-analysis reported average prevalence rates ranging from 0% to 37.5% for children who have experienced any kind of accidental injury (including MVAs, but also other accidental injuries such as burns and sporting injuries), with an average prevalence of 19.82% [3]. Prevalence rates appear to differ according to factors such as the type of injury sustained and in particular, the method of measurement used (e.g. self-report versus diagnostic interview and the use of full or partial diagnostic criteria). For instance, Aaron, Zaglul and Emery [4] reported that 22.5% of participants met criteria for PTSD following a physical injury, although this increased to 47.5% when considering partial PTSD.

Importantly, recent research has indicated little difference in terms of distress and impairment between children meeting full and partial criteria for PTSD [5]. Several alternative approaches have been proposed for the classification of PTSD in youth, which take into account developmental differences in youth experiencing posttraumatic stress. Specifically, Scheeringa and colleagues [6,7] have proposed an alternative PTSD algorithm ("PTSD-AA algorithm"), which removes Criterion A2 (initial response of fear, helplessness or horror) and requires only one re-experiencing symptom, one symptom of avoidance (as opposed to three) and two of hyperarousal. The PTSD-AA algorithm has now been tested in several studies and has been demonstrated to be a better predictor than DSM-IV criteria of psychosocial functioning [7,8]. This suggests that the number of children suffering emotional problems after a traumatic event may be much higher than the prevalence percentages suggested by studies requiring that full diagnostic criteria be met. It also highlights the importance of studying children and adolescents who meet criteria for not only the full PTSD diagnosis, but also the alternative algorithm (PTSD-AA). From this point onwards, the term PTSD will be used to refer to young people experiencing either full PTSD or meeting the PTSD-AA algorithm.

In terms of consequences, PTSD is a chronic and debilitating disorder that is associated with significant impairments in both social and academic functioning [9]. When considering children who have experienced accidental injury specifically, PTSD is also associated with elevated rates of other emotional and behavioural problems (especially anxiety disorders), in comparison to community samples and children admitted to hospital for non-trauma related health reasons [10]. Further, PTSD is also associated with poorer health-related quality of life for children (i.e., the impact of disease and therapy on a person's life situation), both in the short-term and the long-term, including poorer adherence to medical protocols [11].

### Interventions for childhood PTSD

Trauma-focused CBT (TF-CBT) has demonstrated the strongest level of empirical support as the treatment of choice for PTSD in adults. Specifically, models of the psychological impact of trauma suggest that the way in which people remember and recount threatening events significantly affects how well they manage and adjust to those experiences [12]. Similar models of TF-CBT have also been described for childhood PTSD [13]. In the child and adolescent literature, the evidence base is also strongest for TF-CBT interventions [14-16], however the vast majority of research has examined a very narrow sub-group of traumatic events that may affect children and adolescents (e.g. sexual abuse). Unfortunately, accidental injuries represent a sub-group of traumatic events affecting children and adolescents that is far more commonly encountered and yet has received only very little scientific attention. It is possible that this group of sufferers may present with different symptoms to those experiencing ongoing sexual abuse or repetitive trauma and subsequently, may require different treatment approaches [17].

In light of these important differences, it has been suggested that the generalization or application of knowledge gained from treatment outcome studies with young people who have experienced child sexual abuse to young people who have experienced trauma other than abuse may be highly problematic [18]. In more recent years, Cohen and colleagues' TF-CBT has demonstrated efficacy for children who experience PTSD following traumatic events other than sexual abuse, including traumatic grief, domestic violence, terrorism, natural disasters and multiple traumatic events [15]. However, a review of the literature indicates only a handful of controlled trials that have been published examining TF-CBT for children with PTSD following a single-incident trauma.

Chemtob, Nakashima and Hamada [19] and Stein and colleagues [20] provided school-based interventions to children experiencing trauma symptoms following a hurricane and exposure to violence respectively. Both studies concluded that the child-focused interventions evaluated resulted in significant reductions in self-reported PTSD scores. Although extremely important, neither of these studies included a WL condition, nor did they utilise diagnostic status as a primary outcome measure. In a more recent controlled trial, Smith and colleagues [21] compared an individual child-focused CBT condition (in which joint parent-child sessions were carried out as deemed appropriate) to a WL condition, in a sample of 24 young people who met full criteria for PTSD following either an MVA or exposure to violence. They reported that individual TF-CBT was effective in reducing indicators of PTSD in children and adolescents who had experienced a single incident trauma. However, the number of participants was extremely small and the intervention evaluated does not appear to have been uniformly administered to all participants (this is particularly relevant when considering to what extent parents were involved in treatment). Importantly, none of the studies to date appear to have examined health-related outcomes such as physical functioning or adherence to medical protocols.

Overall, research has demonstrated strong support for TF-CBT in the treatment of childhood PTSD, however, controlled investigations of youth exposed to single-incident traumas are lacking. On the limited evidence available, it appears that trauma-focused CBT may be an effective treatment for PTSD in children and adolescents exposed to accidental injuries such as MVAs. However, more controlled trials and empirical evidence is required and several important questions remain unanswered, including the importance of parental involvement in treatment and optimal timing of interventions.

### The role of parents in the treatment of PTSD in children and adolescents

The role of parenting behaviours and parental anxiety have long been recognised as crucial factors in the development and maintenance of childhood anxiety disorders [e.g. [22]]. Recent research also supports the proposal that family-focused CBT results in significantly better long-term outcomes for children with anxiety disorders. For example, Cobham, Dadds, Spence, & McDermott [23] reported that, 3 years after completion of treatment, anxiety-disordered children/adolescents who had received family-focused CBT were significantly more likely to be anxiety diagnosis-free (92%) compared with those who had received child-focused CBT (69%). Increasingly, it is being acknowledged that parental reactions, psychopathology and coping strategies all have the potential to play an important role in the development and maintenance of children's PTSD [e.g. [24,25]].

To date, very few treatment studies in this area have included a parental treatment component, with those that have concluding that a combined parent and child trauma-focused CBT condition results in the best outcomes for children. Although the recent pilot study conducted by Smith et al. [21] did include some degree of parental involvement, this was not quantified and did not appear to be administered in a standardized fashion across participants. From the limited evidence available it is clear that an important direction for future research centres around the question of whether involving parents in treatment significantly enhances child-focused CBT for PTSD.

### The importance of early intervention

Another issue which requires attention concerns the optimal timing for delivery of CBT interventions for childhood PTSD. In terms of the course of PTSD in children and adolescents, the current adult literature and the few existing prospective studies of children presenting with PTSD suggest that a steep decline in PTSD rates may be expected within the first year following the traumatic event [26-28]. However, a significant proportion of children who initially present with PTSD following a traumatic event are highly likely to continue to experience PTSD over the long-term if they do not receive treatment. There is then a fine balance to be struck between the need to provide early intervention in order to prevent the development of emotional and behavioural problems after trauma, and the need to avoid treating young people who do not need treatment and would instead recover on their own.

Strategies for identifying those most at risk for the development or continuation of poor psychological adjustment after trauma may represent the best way forward. Kenardy and colleagues [10] reported that early self-reported symptoms of PTSD in children injured in accidents (measured at 1-2 weeks post-injury) predicted the presence of PTSD symptoms at 4-6 weeks and 6 months post-injury. Using a specially developed scale (the Child Trauma Screening Questionnaire; CTSQ), the diagnosis of 91% of children was correctly predicted in this study. Thus, it is possible to identify those children most likely to be 'at-risk' of PTSD diagnosis within the first two weeks following the injury. Moreover, it is proposed that early intervention targeted at children who demonstrate the presence of PTSD symptoms at 4-6 weeks, will be effective in significantly reducing the longer-term prevalence of PTSD and anxiety disorder symptoms.

Thus, there is a pressing need for a controlled trial examining the efficacy of a trauma focused, CBT early intervention for the treatment of PTSD in children following exposure to accidental injury. Further, a controlled trial examining the impact of CBT on psychosocial as well as health related outcomes is necessary. A trial of this sort provides the opportunity to examine whether trauma-focused CBT interventions are suitable for PTSD resulting from accidental injury, as opposed to repetitive trauma such as sexual abuse or natural disasters. Given the importance of parental involvement in the treatment of other child anxiety disorders, yet the lack of examination of this issue in the treatment of youth PTSD (overall), there is also a need to examine whether CBT interventions can be enhanced through the addition of a parent-based component.

The study protocol presented here provides an overview of the present trial including a description of the methods, design, and current status of the trial as well as a discussion of the possible implications that may arise from the findings.

## Methods/Design

### Design of the Trial

This study is designed as a three arm randomised controlled trial with two active interventions ('*child-focused' *and *'family-focused'*) and one comparison (*'waitlist'*) condition. The study will be conducted across 3 hospitals in Australia, The Royal Children's Hospital (RCH) in Brisbane, The Mater Children's Hospital (MCH) in Brisbane and the Women's and Children's Royal Hospital (WCH) Adelaide. There will be six measurement occasions: screening at 1-2 weeks post-hospital admission, baseline, 4-weeks post-baseline, post-intervention, and follow-ups at 6 and 12 months after the post-intervention assessment. A half crossover design will be used to allow baseline and post-test comparisons between the two active treatment conditions and waitlist control group on primary and secondary outcome measures, as well as increasing the sample size for the final evaluation at follow-up. Thus, all children in the waitlist condition will be randomly allocated to one of the two active treatment conditions following their initial waiting period.

This study was granted ethical approval by the University of Queensland Human Research Ethics Committee (protocol number2008002119), the Royal Children's Hospital (Brisbane) Children's Health Service District Ethics Committee (protocol number HREC/09/QRCH/41), The Mater Health Services (Brisbane) Human Research Ethics Committee (protocol number 1305C) and the Children Youth and Women's Health Services (Adelaide) Human Research Ethics Committee (protocol number REC2149/2/2112). The study is funded by an NHMRC Project Grant (569660). The measures to be administered at each time point are listed in Table 1.

**Table 1:** Measures to be administered at each measurement occasion

Measure	Screening	Baseline	4-weeks post-baseline	Post-test	6-month	12-month
Demographics	X	X				
Child Trauma Screening Questionnaire	X	X	X	X	X	X
Heart-rate	X					
Injury severity	X					
Clinician Administered PTSD Scale-Child and Adolescent Version		X	X	X	X	X
Faces Pain Scale		X		X	X	X
Child PTSD Symptom Scale		X	X	X	X	X
Child Depression Inventory-Revised		X		X	X	X
Spence Children's Anxiety Scale		X		X	X	X
Pediatric Quality of Life Scale - Child version		X		X	X	X
Child Behavior Checklist		X		X	X	X
State Trait Anxiety Inventory		X		X	X	X
Pediatric Quality of Life Scale - Parent version		X		X	X	X
Depression, Anxiety and Stress Scale		X		X	X	X
Posttraumatic stress Diagnostic Scale		X	X	X	X	X
Satisfaction/Credibility Questionnaire				X		
Global Functioning/Improvement Scale			X	X	X	X

### The Interventions

The intervention to be evaluated consists of two integrated but distinct programs. The first program is for parents, "My child and the accident: A story with a good ending" [29] and the second program is for children/adolescents, "Me and the accident: A story with a good ending" [30]. Participants in the '*family-focused' *intervention will receive *both *the child and parent program whereas participants in the *'child-focused' *intervention condition will receive *only *the child program.

These programs were developed and piloted as part of a research project conducted by two of the authors (VC & JK). In both treatment conditions, participants will be seen by a psychologist individually. All sessions in both treatment programs are of approximately 1.5 hours in duration. In the family-focused intervention condition, the parent and child programs will be delivered consecutively, with parents completing parent sessions first. The parent program consists of four sessions and aims to: provide psychoeducation about PTSD, as well as a rationale for the child program; focus on danger perceptions and the way these change after a traumatic incident (with an emphasis on factors such as the possible communication of threat from parent to child and reinforcement of perceptions of danger); focus on changes in parenting practices after a traumatic incident affects a family member and encourage parents to think about their own parenting behaviours and whether these are likely to be helpful or not; and support parents to develop the skills to effectively parent a child experiencing PTSD.

The child/adolescent program consists of six sessions. This program takes a strengths-based, resilience-building approach; is age appropriate, incorporates creative drawing and writing tasks and aims to: provide psychoeducation about the role of thoughts, behaviours (avoidance) & physical reactions in anxiety (and in PTSD in particular); emphasize the importance of the young person's story of his/her accident and their perceptions of current danger or threat; assist children/adolescents in identifying the 'hot spot' thoughts (i.e., particularly emotive thoughts or images) in their story; provide young people with the skills to challenge their hot spot thoughts and to manage their "intruder thoughts" (i.e., any intrusive thoughts or images experienced); and plan for the future (relapse prevention).

### Participants, Recruitment & Inclusion/Exclusion Criteria

Participants will include children and adolescents aged between 7 and 16 years (and their parents). Children who have presented to either the Emergency Department of the hospital wards following an accidental injury (e.g. sporting injury, burns, MVA) will be eligible for inclusion. A complete list of inclusion/exclusion criteria is given in Table 2.

**Table 2:** Inclusion/Exclusion Criteria

Inclusion Criteria	Exclusion Criteria
Aged between 7 and 16 years	Parent's English insufficient for questionnaire completion
Admission to hospital for a minimum of 6 hours for accidental injury	Developmental delay or mental retardation in the child
Endorsement of CTSQ items indicating 'at risk' status within 2 weeks of admission	Moderate to severe head injury or posttraumatic amnesia following the accident
Elevated clinical symptoms defined by meeting PTSD-AA or DSM-IV criteria on the structured diagnostic interview (Clinician-administered PTSD Scale for Children and Adolescents: CAPS-CA; Nader et al.)	Alcohol or substance abuse, or psychosis in the caregiver
Consent to participate in the study	Severe depression or suicide risk in the child
Family must live within 200 km's or be willing to travel for weekly therapy sessions	Child currently under the care of Department of Child Safety
	Injury due to physical or sexual abuse (intentional injury)

Recruitment procedures will be identical across the three hospital sites and will involve several stages.

#### Stage 1 - Participant identification

Within each hospital's Emergency, Medical and Surgical wards, Research Nurses or Research Assistants will identify eligible participants according to inclusion/exclusion criteria. Eligible families will be contacted while still in the hospital and given an Information Sheet and a "Consent to Contact" form.

#### Stage 2 - Screening

Eligible families who have given their permission to be contacted will be contacted either by phone (or in person if the child/adolescent is still in hospital) 1-2 weeks after their initial admission to the hospital. If the family remains interested in participating in the study, consent and assent forms will be completed and the Child Trauma Screening Questionnaire [CTSQ: [10]], a brief 10-item self-report screen, will be administered to the child/adolescent by a trained Research Assistant. Administration of the CTSQ may occur either over the telephone or at the hospital, depending on whether the young person has been discharged. Children/adolescents scoring above the cut-off score (>5) on the CTSQ will be judged to be 'at risk' for developing PTSD and will be invited to proceed to Stage 3.

#### Stage 3 - Diagnostic assessment

At approximately 4-6 weeks following the young person's initial admission to the hospital, those participants judged to be at risk for PTSD will undergo a face-to-face diagnostic assessment. At this time, a diagnostic interview, the Clinician Administered PTSD Scale - Child and Adolescent Version [CAPS-CA; [31]] will be conducted by a trained Research Assistant with children/adolescents. If the participant meets diagnostic criteria for either full or partial PTSD, the measures outlined in Table 1 will be administered to children/adolescents and parents. In order to proceed past this stage, children/adolescents must meet at least 2 out of the 3 cluster criterions for PTSD on the CAPS-CA. In other words, they must meet either full or partial diagnostic criteria for PTSD.

#### Stage 4 - Intervention

Participants who fulfill either full or partial diagnostic criteria for PTSD and agree to participate in the study will be randomly assigned to either the waitlist condition (WLC) or one of the two active treatment conditions; child-focused cognitive-behavioural therapy (CF-CBT) or family-focused cognitive-behavioural therapy (FF-CBT). Participants in the WLC will be randomly assigned to one of the two active treatment conditions after 10 weeks of having been in the WLC (following a re-assessment at this time).

#### Stage 5 - 4-week post-baseline assessment

Participants in the FF-CBT condition will complete the parent program in the first four weeks, and to evaluate changes in parent and child behaviours over this time, a brief assessment will be conducted following the completion of parent sessions, or at 4-weeks post-baseline assessment. To enable a comparison to participants in the CF-CBT and WLC groups, all participants will complete this assessment at 4-weeks post-baseline assessment. Measures to be delivered at this assessment point are outlined in Table 1.

#### Stage 6 - Post-intervention assessment and follow-up

All participants will be followed up at post-intervention and 6 and 12 months following the completion of treatment, using the assessment battery outlined in Table 1 and described below. In addition, as noted earlier, families in the waitlist condition will be re-assessed following completion of the 10-week monitoring period. The Research Assistants involved in conducting pre-treatment and follow-up assessments will be blind to participants' treatment condition.

This study will aim to recruit a total of 140 participants (approximately 45 in each condition). Recruitment will be carried out over an 18-24 month period at three hospital sites. A comparison of the two active treatments to the waitlist control condition will establish the basic efficacy of both CF-CBT and FF-CBT. A comparison of the two active treatment conditions will test whether the efficacy of CBT for PTSD following accidental injury can be enhanced with parent involvement. Importantly, the battery of questionnaires used in this study will allow assessment of whether the active treatment conditions are associated with improvements in psychological symptoms as well as health-related quality of life. Further, the design of the study will allow for an examination of improvements and maintenance of gains (for the active treatments) over a 12-month follow-up period.

### Study Hypotheses

It is hypothesized that:

• Following treatment, compared to participants in the WLC, participants in both active treatment conditions will demonstrate significantly greater reductions in child trauma, anxiety and depressive symptoms, significantly higher ratings on a health-related quality of life measure and significantly reduced ratings of functional impairment.

• Compared to participants in the CF-CBT treatment condition, participants in the FF-CBT treatment condition will demonstrate significantly greater reductions in child trauma, anxiety and depressive symptoms, significantly higher ratings on a health-related quality of life measure, significantly reduced ratings of functional impairment, significantly greater reductions in parental anxiety, trauma and depressive symptoms and significantly increased parental perception of their own ability to support their child.

• These treatment gains (and differences) will be maintained over 6- and 12-month follow-up.

### Primary Outcome Measure

The primary outcome measures will involve an assessment of posttraumatic stress symptoms. Diagnostic status, symptom severity and associated disability/impairment will be assessed using the Clinician Administered PTSD Scale for Children [31].

### Secondary Outcome Measures

Secondary outcome measures include a variety of measures administered to assess child and parent outcomes.

Child secondary outcomes include: trauma symptoms/risk indicators, assessed using the Child Trauma Screening Questionnaire [CTSQ; [10]]; PTSD symptoms, measured through the self-report Child PTSD Symptom Scale [CPSS; [32]]; anxiety, measured by the Spence Child Anxiety Scale [SCAS; [33]]; depression, measured by the Child Depression Inventory [CDI; [34]]; quality of life, measured through the Pediatric Quality of Life Inventory, Child and Parent Versions [PedsQL; [35]]; internalising and externalising symptoms, measured by the Child Behavior Checklist [CBCL; [36]]; intensity of children's pain, measured by the Faces Pain Scale - Revised [FPS; [37]].

Parent secondary outcomes include: parent PTSD symptoms, assessed through the self-report, Posttraumatic Stress Diagnostic Scale [PDS; [38]]; parental psychopathology, assessed by the Depression Anxiety and Stress Scale [DASS; [39]]; parental state and trait anxiety, measured by the State Trait Anxiety Inventory [STAI; [40]].

In addition, overall distress and improvement will also be assessed using a Global Assessment of Impairment/Improvement Scale developed by the authors.

### Subsidiary Outcome Measures

Subsidiary outcomes will be measured. Specifically, satisfaction with the treatment program will be measured through an author developed Program Satisfaction Questionnaire, completed by parents and children following the intervention. Severity of injury will be assessed using the Injury Severity Score [ISS; [41]] and Abbreviated Injury Scale [AIS; [42]].

### Sample Size and Power Calculations

Given the limited research examining trauma-focused CBT interventions for childhood PTSD following accidental injury, calculations of power was based on existing preliminary evidence and related trials. Although research has traditionally demonstrated large effect sizes for the comparison of CBT interventions to no therapy or control conditions in the treatment of child anxiety disorders, there is less reporting of the expected effect size between two active treatments. Previous research conducted by Cobham et al. [23] demonstrated a large effect size (w = 0.50) for the comparison between family and child only intervention for childhood anxiety disorders. Thus, similar differences are anticipated in the present trial.

The study was powered on the comparison between the family-focused versus the child-focused intervention, as this comparison would require the most power whilst also allowing sufficient power to detect differences between the waitlist and active treatments. For the evaluation of diagnostic change (e.g. whether children became free of their PTSD diagnosis), and accounting for an attrition rate of approximately 20%, a sample size of approximately 140 participants in total across the three groups, waitlist, FF-CBT and CF-CBT, will provide sufficient power to detect differences of a large magnitude between family treatment and child only treatment conditions. Greater power will be obtained by randomizing waitlist participants into the two active treatments following the completion of their waiting period.

### Random Allocation Procedure

Randomisation of participants to treatment conditions will be performed by a staff member who is not involved in the recruitment, collection of data or interview procedures. Blocked randomisation is determined using a computer program which generates a list of random numbers and allocation to one of the three conditions. Randomisation will occur immediately following the completion of the baseline interview with the child, with participants informed at this time. Participants will commence their allocated condition one week later.

### Statistical Considerations

Statistical analyses will be performed by a team member who has not been involved in assessing eligibility of participants, allocation of individuals to treatment conditions, administration of treatment, collection of follow-up assessment data or entering of data. Prior to investigating treatment outcome, the three groups will be compared for pre-treatment equivalence on demographic and baseline assessment measures. If significant differences are found, this will be taken into account by including these variables as covariates in outcome analyses. The outcome data will be analysed and reported in terms of statistical significance of differences between groups in change over time on outcome, clinical significance of the change, and effect sizes.

Primary analyses will be undertaken on an intent-to-treat (ITT) basis, including all participants who have been randomized to a condition, regardless of withdrawal from the condition/study. Separate analyses will also be conducted for those who complete treatment. Differences in diagnostic status across groups will be examined using chi-square analyses for each assessment occasion. Continuous data will be analysed using the preferred approach of linear mixed effects modelling, which is able to include participants whose data may be missing at various time points. Other outcomes such as demographic characteristics and reasons for dropout will be described.

## Discussion

This trial represents an opportunity to test the potential benefit of a trauma-focused CBT intervention for the treatment of childhood PTSD following accidental injury (as opposed to other forms of traumatic events). The proposed research is of enormous significance and provides innovations at many points along a clinical care continuum. For the first time, children and adolescents presenting to Australian hospitals following an accidental injury will receive a routine screen of their psychological functioning. Unfortunately, this does not currently occur, despite the well documented adverse psychological consequences associated with exposure to such traumatic events [10,43]. Such screens will facilitate the early identification of young people who may be at risk of developing (or have already developed) PTSD.

The proposed research aims to use a stepped screening and assessment approach to identify those young people most at risk of maintaining a diagnosis of PTSD over time. This represents a particularly important step forward in terms of indicated early intervention, whereby we are able to accurately identify and then treat those children/adolescents who are at greatest risk of the very significant repercussions of PTSD. At a broad level then, this research has the potential to promote a new model of service provision and improve the efficiency of care provided through the hospital systems to these young people.

The current research also provides an opportunity to examine treatments that focus on both full and partial PTSD in children/adolescents following an accidental injury. To date, no treatment studies (let alone an RCT) have been conducted exclusively with this population (although one pilot RCT has included accidental injuries). In addition, the present research provides an opportunity to examine the importance of incorporating a family component within such interventions. The design of the study allows for an examination of whether child or family-focused interventions are associated with greater improvements, which to date, has not been evaluated in a systematic way with children and adolescents experiencing PTSD following any sort of single-incident trauma. Conducting an RCT with this population represents a significant advancement of scientific knowledge in this field. In addition, in relation to the follow-up of treated participants, the proposed research will be the first RCT with children experiencing PTSD following an accidental injury to include a 12-month follow-up point.

Finally, although the primary focus (in terms of outcomes) of this research is psychiatric (i.e., trauma, depressive and anxiety symptomatology), this study provides an opportunity to also examine outcomes relating to physical health. Specifically, it allows an examination of whether children's and parents' perceptions of children's health-related quality of life and physical functioning are improved following the two active interventions. If effective in improving children's psychological well-being, physical health, treatment adherence and quality of life, these interventions may have significant benefit for improving medical and psychological outcomes in the population as a whole.

In summary, the major benefits of the proposed research will be to provide a more structured psychological care pathway for children/adolescents presenting with accidental injuries, and to create an evidence-base for easily disseminated psychological interventions designed to meet the needs of the significant number of Australian children and adolescents who experience adverse emotional effects following an accidental injury.

## Status of the Trial

The study commenced in February, 2010. Participants will be recruited during an 18-20 month period, from February, 2010 to September, 2011. The trial is expected to end in December, 2012.

## Competing interests

The authors declare that they have no competing interests.

## Authors' contributions

JK, VC, RN, and BM developed the trial protocol and wrote the applications for NHMRC Grant 569660. JK and SM further developed the details of the trial protocol. SM drafted the manuscript. All authors contributed to the editing of the manuscript. All authors have read and approved the final manuscript.

## Author information

**JK**. Professor Kenardy has specific expertise in the psychological impact of trauma in children and adolescents. He also has extensive experience in the design and execution of clinical trials of psychological interventions.

**VC**. Dr Cobham has specific expertise in the psychological impact and treatment of anxiety disorders (including PTSD) in children and adolescents. As the primary author of the interventions, she will provide clinical expertise to the project in supervising therapists and overseeing the provision of the interventions. She also has specific expertise in the design and execution of clinical trials of psychological interventions.

**RN**. Associate Professor Nixon's primary area of expertise is in the etiology, maintenance and treatment of child and adult posttraumatic stress disorders.

**BM**. Associate Professor McDermott has investigated and published on the effects of single-event trauma following natural disasters and been involved in design of child and adolescent PTSD interventions.

**SM**. Trial Manager for the trial and Postdoctoral Research Fellow with Professor Justin Kenardy. She also has expertise in the execution of clinical trials of psychological interventions for child anxiety.

## Pre-publication history

The pre-publication history for this paper can be accessed here:

http://www.biomedcentral.com/1471-244X/10/92/prepub
