# Protocol for a randomised controlled trial of risk screening and early intervention comparing child- and family-focused cognitive-behavioural therapy for PTSD in children following accidental injury

**DOI:** 10.1186/1471-244X-11-15

**Published:** 2011-01-20

**Authors:** Justin Kenardy, Vanessa Cobham, Reginald DV Nixon, Brett McDermott, Sonja March

**Affiliations:** 1 Centre of National Research on Disability and Rehabilitation Medicine, School of Medicine, University of Queensland, Herston QLD 4029, Australia; 2 School of Psychology, University of Queensland, St Lucia QLD 4072, Australia; 3 Child and Youth Mental Health Service, Mater Children's Hospital, Annerley Road, South Brisbane QLD 4101, Australia; 4 School of Psychology, Flinders University, Adelaide SA 5001, Australia

## Correction

After the publication of this article [1], we became aware of the fact that there was an error in Table 1 "Measures to be administered at each measurement occasion". This correction contains the revised Table. Specifically, within Table 1 it should be noted that the Child Trauma Screening Questionnaire (CTSQ) and Clinician Administered PTSD Scale-Child and Adolescent Version (CAPS-CA) are *only *administered at screening, baseline, post-test, 6-month and 12-month follow-up.

**Table 1:** Measures to be administered at each measurement occasion

Measure	Screening	Baseline	4-weeks post-baseline	Post-test	6-month	12-month
Demographics	X	X				
Child Trauma Screening Questionnaire	X	X		X	X	X
Heart-rate	X					
Injury severity	X					
Clinician Administered PTSD Scale-Child and Adolescent Version		X		X	X	X
Faces Pain Scale		X		X	X	X
Child PTSD Symptom Scale		X	X	X	X	X
Child Depression Inventory-Revised		X		X	X	X
Spence Children's Anxiety Scale		X		X	X	X
Pediatric Quality of Life Scale - Child version		X		X	X	X
Child Behavior Checklist		X		X	X	X
State Trait Anxiety Inventory		X		X	X	X
Pediatric Quality of Life Scale - Parent version		X		X	X	X
Depression, Anxiety and Stress Scale		X		X	X	X
Parent Diagnostic Scale		X	X	X	X	X
Satisfaction/Credibility Questionnaire				X		
Global Functioning/Improvement Scale/			X	X	X	X

We also note that there was an inconsistency between text in the introduction and methods section. The term PTSD is used in this article to refer to young people experiencing either full PTSD (meeting DSM-IV criteria) or meeting the alternative PTSD-AA algorithm (PTSD-AA) [2,3]. Thus, within the Methods section describing Participants, Recruitment and Inclusion/Exclusion Criteria, it should be clarified that children/adolescents will progress beyond Stage 3 (Diagnostic assessment) if they meet DSM-IV *or *PTSD-AA criteria for PTSD on the CAPS-CA.

## Pre-publication history

The pre-publication history for this paper can be accessed here:

http://www.biomedcentral.com/1471-244X/11/15/prepub
